# Effect of ACASI on Reporting of Abortion and Other Pregnancy Outcomes in the US National Survey of Family Growth

**DOI:** 10.1111/sifp.12068

**Published:** 2018-07-24

**Authors:** Laura Lindberg, Rachel H. Scott

## Abstract

Abortion is a behavior that is stigmatized and difficult to measure. To improve reporting of abortion and other sensitive behaviors in the United States, the National Survey of Family Growth (NSFG) supplements the interviewer administered face‐to‐face (FTF) interview with audio computer‐assisted self‐interviewing (ACASI). This paper estimates differential reporting of abortion and other pregnancy outcomes (miscarriage, live birth) in the NSFG (2002, 2006–2010, 2011–2015) between women's ACASI and FTF interviews. Examining reporting of less stigmatized pregnancy outcomes can help understand the relative contributions of stigma and survey‐level factors in reporting of abortions. More women reported abortions, miscarriages and births in the ACASI than the FTF interview. Differences in reporting were moderated by the length of recall. The ACASI elicited relatively more reporting of abortions and miscarriages among non‐white and low‐income women. Reporting ratios increased over time. ACASI is a tool that may work differently across time, for different measures, and with varying survey contexts.

## INTRODUCTION

Demographic research is constrained by the challenges of measuring sensitive behaviors, such as abortion, in surveys. Individuals may deliberately misreport stigmatized behaviors in order to provide more socially‐desirable responses (Groves et al. 2011; Tourangeau, Rips, and Rasinski 2000). Incomplete and inaccurate abortion estimates create challenges for monitoring of trends, levels and differential patterns of abortion (Sedgh et al. 2016), abortion safety (Ganatra et al. 2017), and other fertility‐related outcomes such as intended and unintended pregnancy (Bearak et al. 2018; Sedgh, Singh, and Hussain 2014; Singh, Sedgh, and Hussain 2010) and contraceptive failure rates (Polis et al. 2016). These challenges in abortion measurement occur in settings in which abortion is illegal (Singh, Remez, and Tartaglione 2010) as well as legal but socially sensitive (Houzard et al. 2000; R. Jones and Kost 2007; Moreau et al. 2004; Scott et al. 2017, 2018).

Even in the United States where abortion is legal, it remains socially stigmatized, described by Norris et al as “the discrediting of individuals as a result of their association with abortion” (Norris et al. 2011). This stigma is theorized to influence individuals’ willingness to report their abortion experiences in surveys (Astbury‐Ward, Parry, and Carnwell 2012; Tourangeau, Rips, and Rasinski 2000). Multiple studies have found that compared to external surveillance counts, women underreport abortion in the National Survey of Family Growth (NSFG), the premier survey of fertility behaviors in the US (Fu et al. 1998; E. Jones and Forrest 1992; R. Jones and Kost 2007; Tennekoon 2017), as well as in other US surveys (Jagannathan 2001; E. Jones and Forrest 1992; Tierney 2017; Udry et al. 1996). Jones and Kost (R. Jones and Kost 2007) estimate that 47 percent of abortions are reported in the 2002 NSFG; Tennekoon (2017) estimates that in the 2006–13 NSFG, 35 percent of abortions are reported.

Efforts to improve survey measurement of abortion and other sensitive behaviors have focused on methods of reducing the sensitivity of reporting, including approaches such as changing question wording, changing the placement of the questions within the survey, and increasing the privacy felt by the respondent (Tourangeau and Yan 2007). The use of audio computer‐assisted self‐interviewing (ACASI) has become widely adopted in surveys in the US and worldwide^1^; ACASI allows respondents to answer pre‐recorded questions on a computer which the interviewer does not see, providing enhanced privacy and confidentiality as compared to interviewer administered face‐to‐face (FTF) questions. ACASI has been demonstrated to improve the reporting of many self‐reported sensitive behaviors in both the US and international settings and there is increasing attention being given to using technology as a tool to improve reporting (Gnambs and Kaspar 2015; Hewett et al. 2008; Langhaug, Sherr, and Cowan 2010; Mensch et al. 2008; Phillips et al. 2010; Tourangeau and Yan 2007).

To improve reporting of abortion as well as other sensitive behaviors, since 1995 the NSFG has supplemented the FTF interview with ACASI (J. Kelly et al. 1997). Prior NSFG studies have documented more women reporting abortions in the ACASI as compared to the FTF. For example, 15 percent more women reported ever having an abortion in the ACASI than the FTF interview in unweighted analyses of the 2002 NSFG (Tourangeau, Rips, and Rasinski 2000) and in the 1995 survey, the ACASI resulted in 29 percent more women reporting an abortion than in the FTF interview (Peytchev 2012). These increases in reporting in ACASI have been interpreted as more complete and accurate than the FTF interview measures, although they have still been incomplete as compared to external surveillance counts collected directly from abortion providers (Fu et al. 1998; R. Jones and Kost 2007).

In this analysis, we focus on patterns of abortion reporting by survey mode in the NSFG to directly test the assumption that ACASI improves reports of abortion by increasing privacy. We examine alternate mechanisms for differences in reporting between the ACASI and FTF interview, including differences in question wordings and recall periods that may impact the cognitive demands of the survey mode, that is to say the cognitive processing that is required to answer the question (Groves et al. 2011), and possible changes over time. To better understand the role that ACASI plays in abortion reporting, we also examine patterns of reporting of other, less stigmatized pregnancy outcomes. Prior research suggests that for women, abortion, spontaneous miscarriage, and live births are differentially sensitive (Groves et al. 2011; Cowan 2014), suggesting that patterns of reporting across survey modes may vary across these pregnancy outcomes. Identifying differences and similarities in the patterns of reporting of abortion and other pregnancy outcomes can help us understand the role of stigma and other social and survey‐level factors in abortion reporting. This understanding could inform efforts to improve measurement of abortions in surveys, and illuminate efforts to collect abortion data directly from women. These findings may also be relevant to the survey measurement of other sensitive behaviors such as sexual behavior, illicit drug use, or violence (Kalsbeek and Carol 2014; C. Kelly et al. 2014; Turner et al. 1998).

## BACKGROUND AND HYPOTHESES

### Survey Measures

As discussed above, the NSFG collects data on pregnancy outcomes in both the FTF and ACASI, allowing for comparisons of reporting across survey modes. Women first provide a full pregnancy history in the FTF interview. Women are asked how many times they have ever been pregnant, and are subsequently asked further detailed questions about each pregnancy in turn, including its outcome (live birth, still birth, abortion or miscarriage) and the date that the pregnancy ended. After completing the full FTF interview, women receive the ACASI in which they are asked, with a single, separate question for each outcome, to report the number of live births, abortions and miscarriages they have had within a specified time period (lifetime in 2002, last five years in later rounds). The specific question wordings are shown in Table 1; the full survey instruments are available at https://www.cdc.gov/nchs/nsfg/nsfg_questionnaires.htm.

**Table 1:** Question wording of pregnancy outcomes items, by year of interview and survey, National Survey of Family Growth

2002	2006–2010, 2011–2015
Face‐to‐Face Interview	
In which of the ways shown on this Card did the pregnancy end?	
(1) Miscarriage	
(2) Stillbirth	
(3) Abortion	
(4) Ectopic or Tubal Pregnancy (coded as Miscarriage)	
(5) Live birth by Cesarean section	
(6) Live birth by vaginal delivery	
ACASI	
INTRO_J5 INTRO‐J5. The information you provide about the outcome of any pregnancies you may have had is very important for this study. Sometimes women who take part in the study are reluctant to tell an interviewer about some of their pregnancies. Remember, your interviewer will not know how you answer these questions, and will not ask you any questions about your answers. Please press [Enter] to continue.	INTRO_J5 JB_0. Sometimes women are reluctant to tell an interviewer about some of their pregnancies, especially those pregnancies that ended in abortion or with babies they no longer live with. In the next set of questions, please give a complete count of all your pregnancies, even if you did not mention them all to the interviewer.
JB‐1: First, are you pregnant now?	
JB‐2: How many pregnancies have you had that resulted in live birth, that is, a baby born alive? Having twins or triplets should be counted as one pregnancy	JB‐1: Between January (year of interview – 5) and December (year of interview – 1), how many pregnancies did you have that resulted in live birth, that is, in a baby born alive? Having twins or triplets should be counted as one pregnancy
JB‐3: How many pregnancies have you had that ended in miscarriage, stillbirth, or ectopic pregnancy?	JB‐2: Between January (year of interview – 5) and December (year of interview – 1), how many pregnancies did you have that ended in miscarriage, stillbirth, or ectopic pregnancy?
JB‐4: How many pregnancies have you had that ended in abortion?	JB‐3: Between January (year of interview – 5) and December (year of interview – 1), how many pregnancies did you have that ended in abortion?
JB‐5: Based on these last four questions, you have been pregnant (TOTAL BASED ON CASITOTP) times. Is this correct?	
JB‐6: Please think of all the times you have been pregnant, whether you are currently pregnant or the pregnancy ended in live birth, miscarriage, stillbirth, abortion, or ectopic pregnancy? How many times have you been pregnant in your life?	

### Pregnancy Outcome

Our central hypothesis is that ACASI will increase reporting of more sensitive pregnancy outcomes, as compared to the FTF interview. Because of the different levels of sensitivity and stigma surrounding pregnancies ending in abortion, miscarriage and live birth, we also expect patterns of reporting by survey mode to vary across the three pregnancy outcomes. Given the heightened stigma of abortion, we expect more women to report abortions in the ACASI as compared to the FTF interview. Furthermore, the increased reporting in the ACASI is expected to be larger for abortion than the other outcomes. Although women's miscarriage experiences have received less substantive and methodological research attention than abortion, there are indications that they may also be sensitive for women (Bardos et al. 2015; Groves et al. 2011; Cowan 2014), thus, ACASI is expected to increase reporting of miscarriage as well. In the 2002 NSFG, R. Jones and Kost (2007) estimated that the lifetime number of miscarriages reported in the ACASI was 25 percent higher than in the FTF interview. In contrast, women's births have not been considered a sensitive outcome or difficult to measure; the FTF interviews in the NSFG have been estimated to produce birth counts for women comparable to those of vital records (Chandra et al. 2005). Thus, we expect that reporting of births is relatively unaffected by sensitivity bias, so that the ACASI should not increase birth reporting as compared to the FTF interview.

### Respondents’ Characteristics

We hypothesize that the influence of the ACASI on reporting may vary by respondents’ characteristics. The stigma surrounding abortion may vary by social groups, and there is mixed evidence that certain sub‐groups are more likely to perceive or internalize stigma than others (Cockrill et al. 2013; Shellenberg and Tsui 2012). In addition, individuals with greater social resources may be more able to distance themselves from dominant social norms (Bajos and Marquet 2000). Analyses which compare abortion reporting in the NSFG to external surveillance counts have found that women of color and those with low income were among the least likely to fully report their abortions in the FTF interview (Fu et al. 1998; Tourangeau, Rips, and Rasinski 2000). This suggests that the relative privacy afforded by the ACASI may be more important in eliciting disclosure of abortion or miscarriage among these groups. In the 2002 NSFG, ACASI appeared to improve reporting of abortion among black and Latina women but not among non‐Latina white women, but differential reporting by other characteristics was not examined (Tourangeau, Rips, and Rasinski 2000).

### Retrospective Reporting Period

Thus far, we have only considered mode effects in considering differences between reporting in the ACASI and FTF interviews, focusing on how the use of the ACASI may influence reporting by affecting the perceived privacy and confidentiality of the survey experience. However, in the NSFG, the question items about pregnancy outcomes in the FTF and ACASI interview are not precisely the same, and these differences may influence reporting separately from the privacy factor (or may interact with the privacy factor). In other words, other cognitive processes in survey response may influence differential patterns of reporting between the ACASI and FTF interviews.

We hypothesize that differences by survey mode may be due to misdating or telescoping of pregnancies, a phenomenon that occurs when a respondent (intentionally or unintentionally) imports an event into the wrong reference period (Bradburn, Rips, and Shevell 1987). In each round of the NSFG, the FTF interview collected a lifetime pregnancy history with dates of each pregnancy outcome. However, while in 2002 women in the ACASI were asked about their lifetime pregnancies, from 2006 onwards they were asked only about pregnancies occurring in the five years preceding the survey. Asking about this five‐year period was intended to facilitate comparisons with external data sources, but it may also lead to the misdating of pregnancies relative to this five‐year period. We hypothesize that some of the increased reporting in ACASI may be due to incorrect recall of dates. Thus, these changes in the NSFG survey instruments over time can help to distinguish between reporting patterns due to increased privacy versus other recall influences.

### Time Period

The social and political climate surrounding abortion has become more hostile over the thirteen years for which we have comparable data (R. Gold and Nash 2012; Nash et al. 2016), which may have increased the stigma felt by women when disclosing an abortion. ACASI would thus remain important in its ability to conceal survey response from both the interviewer and anyone else who may be in the home at the time. Over time, general distrust of the survey experience may have increased; well‐documented national trends toward declining survey response rates may indicate less trust in the survey experience more generally (Brick and Williams 2013). In this context, the increased privacy of the ACASI may play a larger role over time in encouraging survey responsiveness in the reporting of outcomes too sensitive to report in the FTF interview (Peytchev, Peytcheva, and Groves 2010). In addition, as declining participation rates are often accompanied by attempts to boost it through increased refusal conversion efforts, this may have led to an increase in the proportion of “reluctant” respondents for whom the ACASI may also play a larger role in encouraging disclosure (Fricker and Tourangeau 2010).

In the NSFG data, time period is confounded with the length of retrospective recall, since the ACASI reference period for pregnancies changed from lifetime measures to a five‐year period after 2002. Comparing reporting patterns by pregnancy outcomes can help triangulate some of this confounding, since the sensitivity of reporting births is not expected to have changed over time.

### Hypotheses

To summarize, we propose the following hypotheses.

1. There will be increased reporting of abortion and miscarriage in the ACASI as compared to the FTF interview, but little to no increase in reporting of births.
2. The magnitude of differences in reporting by survey mode will vary by women's socio‐demographic characteristics: age group, race, poverty level, marital status, and religious service attendance.
3. Misdating will explain some of the increased reporting of pregnancies in the ACASI compared to the FTF interview in 2006 and later.
4. Increases in reporting of pregnancy outcomes in the ACASI compared to the FTF interview will be greater in 2006–2010 and 2011–2015 than in 2002.

## DATA AND ANALYSIS

### Data

We draw on data from female respondents in several rounds (2002, 2006–2010, 2011–2015) of the National Survey of Family Growth (NSFG), a periodic national probability household survey of women and men aged 15–44 years in the United States (Groves et al. 2005; Tourangeau, Rips, and Rasinski 2000). The NSFG used a multistage, stratified clustered sampling frame. In‐person interviews in respondents’ homes were conducted in 2002 and then continually from June 2006 to December 2010 and again from June 2011 to June 2015. Participants provided signed consent and received a $40 incentive. The content focused on fertility, contraception, and union formation. Methods of data collection were reviewed by the National Center for Health Statistics’ (NCHS) Institutional Review Board protections for human subjects. Release of the public use data file was reviewed and approved by the NCHS Disclosure Review Board.

### Analysis

We treat the sample as a within subjects design, in which each respondent is assigned to two reporting modes, ACASI and FTF, and test for differences in reporting between the modes. We identified the number of respondents reporting each pregnancy outcome (abortion, miscarriage, live birth) occurring in the same time period in the ACASI and the FTF interview, and then calculate a ratio of these reports. This reporting ratio expresses discrepancies in the proportion of respondents reporting a specific pregnancy outcome in both survey modes. A reporting ratio equal to 1.00 indicates that the same number of respondents reported each pregnancy outcome in the FTF and ACASI interview, while values greater than 1.00 indicate that more respondents report the pregnancy outcome in the ACASI than in the FTF interview. In the 2006–2015 surveys, we also estimated “expanded” reporting ratios comparing pregnancies reported in the last five years in the ACASI to those reported up to six years prior in the FTF calendar data. This allows us to consider if some of the reporting differences between survey modes is due to recall of dates.

We examine differences in the overall reporting ratios between the 2002, 2006–2010 and 2011–2015 surveys to examine changes in the relative effectiveness of the ACASI (as compared to the FTF interview) over time, as well as reporting patterns associated with different recall periods. We also estimate reporting ratios separately by respondents’ social and demographic characteristics including age, education, union status, race/ethnicity, household poverty level, and religiosity for each time period.

When possible, we calculated bootstrapped confidence intervals around the reporting ratios, re‐sampling the data over 5,000 replications. To determine statistical significance within reporting ratios for each pregnancy outcome, we created a distribution of differences between the bootstrapped ratios and tested whether the values from the 2.5th to the 97.5th percentiles overlapped with zero. Bootstrapping is a statistical method for estimating an estimator, for example a standard error, using resampling with replacement (Carpenter and Bithell 2000). It is often used in instances where no established formula for estimating a standard error exists, in this case for a ratio. This bootstrapping approach was not adequate for testing for statistical significance in the reporting ratios between outcomes, and between the standard and expanded ratio, and instead we identify if the ratios have non‐overlapping confidence intervals, a relatively conservative test of significance. We used unweighted data, which is appropriate given that we were testing reporting differences within the sample, and not making generalizations about population‐level prevalence of the pregnancy outcomes or comparing to an external benchmark. Sensitivity analyses conducted, using the survey weights provided by the National Center for Health Statistics, did not reach substantially different conclusions.

## RESULTS

### Analytical Sample

Table 2 reports on sample characteristics for female respondents in the three rounds of NSFG data. Overall, there were 7,643 female respondents in 2002, 12,272 in 2006–2010 and 11,297 in 2011–2015. The unweighted distribution of the sociodemographic characteristics show at least 1000 respondents in each category, except for Other (Non‐Latina), allowing for robust subgroup analyses.

**Table 2:** Sample characteristics for women in the National Survey of Family Growth, by survey year: 2002, 2006–2010, 2011–2015

	**2002**		**2006–2010**		**2011‐2015**	
	N	%	N	%	N	%
Total	7643	100	12272	100	11297	100
Age group						
15–24	2513	33	4382	36	3960	35
25–34	2651	35	4413	36	4128	37
35–44	2479	32	3477	28	3209	28
Race/Ethnicity						
White (Non‐Latina)	4139	54	6301	51	5285	47
Black (Non‐Latina)	1530	20	2535	21	2420	21
Other (Non‐Latina)	385	5	720	6	743	7
Latina	1589	21	2723	22	2852	25
Poverty level income						
0–99%	1606	21	3361	27	3900	35
100–299%	3019	40	5156	42	4231	37
>300%	3018	39	3762	31	3169	28
Marital status						
Currently married	3080	40	3971	32	3410	30
Previously married	1046	14	1563	13	1443	13
Never married	3517	46	6745	55	6444	57
Religious service attendance						
Less than once a month/ Never	3772	49	6006	49	5632	50
Once a month or more	3856	51	6262	51	5661	50

### Overall Patterns of Reporting

Table 3 shows women's overall reporting ratios for each pregnancy outcome in the 2002, 2006–2010 and 2011–2015 NSFG, and the expanded ratios in the latter surveys. As hypothesized, overall more women reported an abortion in the ACASI than the FTF interview in each survey round, as indicated by reporting ratios greater than 1.00. Additionally, more women reported a miscarriage in the ACASI than the FTF in each survey round. In each survey round there was no significant difference in the reporting ratios for abortion and miscarriage, as evidenced by overlapping confidence intervals. The reporting ratio for births was 1.00 in 2002, while about 10 percent more women reported a live birth in the ACASI than the FTF interview in the later survey years. The reporting ratio was significantly smaller for births than abortion or miscarriage in every survey year, with non‐overlapping confidence intervals between outcomes.

**Table 3:** Ratios of number of women reporting abortions, miscarriages, and births in ACASI and FTF modes by survey round: 2002, 2006–2010, and 2011–2015 National Survey of Family Growth

	Abortions			Miscarriages			Births		
	Ratio	95% CI	Expanded Ratio	Ratio	95% CI	Expanded Ratio	Ratio	95% CI	Expanded Ratio
**Survey Period**									
2002	1.14	(1.12–1.16)	1.14	1.12	(1.11–1.14)	1.12	1.00	(1.00–1.01)	1.00
2006–2010	1.32^a^	(1.26–1.39)	1.24^a^	1.41^a^	(1.37–1.45)	1.34^a^	1.10^a^	(1.09–1.12)	1.04^a^
2011–2015	1.46^a^^,^ b	(1.36–1.56)	1.36^a^, ^b^	1.53^a^^,^ b	(1.47–1.59)	1.44^a^, ^b^	1.13^a^^,^ b	(1.11–1.15)	1.06^a^^,^ b

a p < 0.05, reference category is 2002

b p < 0.05, reference category is 2006–2010

Expanded reporting ratios compare pregnancies reported in the last five years in the ACASI to those reported up to six years prior in the FTF calendar data

For each pregnancy outcome, the size of the reporting ratios increased between 2002 and the later survey years for each of the pregnancy outcomes between 2006–2010 and 2011–2015. Given that births had a reporting ratio of 1.00 in the 2002 survey, the elevated reporting ratios for births in the later surveys provides support for the hypothesis that the length of the retrospective recall period influences reporting, even when the behavior is not particularly sensitive.

It appears that the magnitude of the increase in the reporting ratio over time was larger for abortion and miscarriage than for births, suggesting that the reporting of these outcomes might also be influenced by increased sensitivity over time as well as length of recall. Triangulating the patterns between survey years and pregnancy outcome helps to distinguish reporting patterns due to the confounding influences of length of recall (lifetime versus five years) and increased sensitivity over time.

The results of the expanded reporting ratio provide further support for the hypothesis that the misdating of events would explain some of the increased reporting of pregnancies in the ACASI compared to the FTF interview in 2006 and later. For each pregnancy outcome, the size of the reporting ratios was reduced when the expanded measure was examined, contrasting reports in the last 5 years in the ACASI with reports in the last 6 years in the FTF interview; these differences were significant for births, but had small overlaps in the confidence intervals for abortion and miscarriage. Yet, even in these expanded ratios, ACASI still results in more women reporting each pregnancy outcome than the FTF interview.

As a sensitivity analysis, we examined the individual level reporting patterns in the 2011–2015 survey. These suggested that in many cases, women were reporting their lifetime number of births or abortions in the ACASI when asked to report on events in the last five years. We identified women who reported a birth or abortion in the last five years in the ACASI but reported none in this same period in the FTF interview. Of this group, 60 percent of the women with a birth, and 25 percent of women with an abortion reported the same number of births or abortions in the ACASI as they did in their lifetime reports in the FTF interview (results not shown).

### Reporting Ratios in 2011–2015: Subgroup Differentials

Table 4 displays the reporting ratios for each pregnancy outcome by women's characteristics in 2011–2015, comparing the proportion of women reporting each outcome in the last five years between the ACASI and the FTF interview. As noted before, overall, more women reported a pregnancy in the ACASI than in the FTF interview regardless of the outcome, as evidenced by ratios greater than 1.00 for abortions, live births and miscarriages. The abortion reporting ratios varied significantly by women's characteristics in the 2011–2015 NSFG. As hypothesized, Latinas and poorer women had significantly larger abortion reporting ratios than white and wealthier women respectively; both groups previously had documented underreporting as compared to external counts (Tourangeau, Rips, and Rasinski 2000). The abortion reporting ratio for black, non‐Latina women was not statistically significant from that for white women. In addition, the abortion reporting ratios were significantly larger among women aged 35 or older compared to 25–34 year‐olds, but comparatively lower among 15–24 year‐olds, as well as larger among currently married as compared to both previously married and never married women. Women with more frequent religious attendance had significantly higher abortion reporting ratios than those with rarer attendance.

**Table 4:** Ratios of number of women reporting abortions, miscarriages, and births in the last five years in ACASI to FTF modes, by women's characteristics: 2011–2015 National Survey of Family Growth

	Abortions		Miscarriages		Births	
	Ratio	95% CI	Ratio	95% CI	Ratio	95% CI
Total	1.46	(1.36–1.56)	1.53	(1.47–1.59)	1.13	(1.11–1.15)
Age group						
15–24	1.23^a^	(1.14–1.31)	1.41	(1.31–1.52)	1.01^a^	(0.99–1.03)
25–34 *(ref)*	1.46	(1.34–1.58)	1.51	(1.44–1.59)	1.08	(1.06–1.1)
35–44	2.12^a^	(1.66–2.57)	1.66	(1.5–1.81)	1.36^a^	(1.31–1.42)
Race/Ethnicity						
White (Non‐Latina) *(ref)*	1.31	(1.19–1.44)	1.32	(1.26–1.38)	1.12	(1.09–1.15)
Black (Non‐Latina)	1.43	(1.31–1.56)	1.64^a^	(1.5–1.79)	1.22^a^	(1.18–1.26)
Other (Non‐Latina)	1.62	(1.06–2.17)	1.48	(1.26–1.7)	1.06^a^	(1.01–1.11)
Latina	1.65^a^	(1.45–1.86)	1.85^a^	(1.68–2.02)	1.1	(1.07–1.13)
Poverty level income						
0–99%	1.56^a^	(1.4–1.72)	1.67^a^	(1.57–1.77)	1.12^a^	(1.1–1.15)
100–299%	1.50^a^	(1.37–1.63)	1.52^a^	(1.43–1.61)	1.16^a^	(1.13–1.2)
>300% *(ref)*	1.21	(1.1–1.32)	1.31	(1.21–1.4)	1.1	(1.07–1.13)
Marital status						
Currently married *(ref)*	2.28	(1.82–2.75)	1.35	(1.27–1.43)	1.09	(1.07–1.11)
Previously married	1.39^a^	(1.18–1.6)	1.71^a^	(1.55–1.88)	1.31^a^	(1.25–1.37)
Never married	1.33^a^	(1.26–1.41)	1.64^a^	(1.53–1.75)	1.12^a^	(1.1–1.15)
Religious service attendance						
Less than once a month/ Never *(ref)*	1.32	(1.24–1.4)	1.49	(1.4–1.58)	1.13	(1.11–1.16)
Once a month or more	1.69^a^	(1.49–1.88)	1.56	(1.47–1.65)	1.13	(1.1–1.16)

a p < 0.05

(ref) indicates the reference category for each variable

Both black and Latina women were significantly more likely than white women to report a miscarriage in the ACASI than the FTF interview, while the reporting ratio for births was significantly larger among black than white women. For both miscarriage and births, the reporting ratios were significantly larger among those with lower income compared to wealthier women, and significantly smaller among married women than those previously or never married. The reporting ratio for births increased significantly by age; the pattern for miscarriages was similar but not statistically significant. Neither miscarriages nor births had different reporting patterns by women's religious attendance.

The patterns of sociodemographic differentials in reporting are very similar between the 2011–2015, 2006–2010 and 2002 NSFG, despite changes in the question design and length of the retrospective recall period (see Appendices 1 and 2). The one exception is the pattern of abortion reporting among women age 35–44. In the 2006–2010 survey, older women are more than twice as likely to report an abortion in the ACASI than the FTF interview during the five‐year recall period; this reporting ratio is significantly larger than among women younger than age 35. In contrast, in the 2002 NSFG, when a lifetime recall period is used in the both the ACASI and the FTF interview, there are no differences by age in the reporting ratios for births.

## DISCUSSION

ACASI has been adopted widely as an important survey tool for eliciting increased reports of sensitive or stigmatized behaviors. Our analysis focused on the effect of ACASI in the NSFG on reporting of pregnancy outcomes, comparing the number of respondents reporting abortion, miscarriage and live births in both the ACASI and the FTF interview. We found variation in the patterns of reporting by survey mode according to type of pregnancy outcome, respondent characteristics, time period, and the length of the retrospective reporting period. These findings point to the importance of recognizing that the ACASI is a tool that may work differently across time, for different measures, and with varying survey contexts.

### Privacy

Echoing earlier research, we found that more women reported abortions in the ACASI than the FTF interview in each round of the NSFG, suggesting that the additional privacy and perceived confidentiality afforded by the ACASI makes them feel more able to disclose this stigmatized pregnancy outcome. More women also reported miscarriages in the ACASI than the FTF survey, supporting the interpretation that like abortion, miscarriages are a sensitive pregnancy outcome. This sensitivity may not share the same social stigma as abortion, but be related to other psychosocial aspects of the fertility loss (Bardos et al. 2015).

We also found that certain subgroups of women were more responsive to the increased privacy of the ACASI than others. In particular, variations in reporting of abortions and miscarriages by race/ethnicity and income supported the hypothesis that respondents with greater social and economic resources may feel less threatened by social stigma. For some women, a birth may also be sensitive or stigmatized, and the ACASI facilitates reporting; this might explain the elevated reporting ratio for births among previously married women, for example. The ACASI also appeared to facilitate reporting of births among black women, for whom gender and racial inequality have meant, and continue to mean, greater surveillance and regulation of their fertility and reproductive autonomy, particularly for low‐income black women (Roberts 2000).

### Time Period

The estimated increases over time in the reporting ratios for abortion and miscarriage suggest that the confidential nature of the ACASI has become more important in the survey process. As abortion rates in the US decline, the composition of women who have abortions may be shifting toward those for whom it is a more sensitive event, or women may have less exposure to others with abortion experiences, making it feel more stigmatized (Cowan 2014). The ACASI mode may also increase in importance because of an overall decreased willingness to participate in surveys; as the sample contains a greater proportion of “reluctant” respondents, there may be greater distrust of the survey process and less willingness to report sensitive behaviors in the FTF interview (Groves 2006). In one study, women with a lower likelihood of participating in the NSFG were more likely to report an abortion in the ACASI and not the FTF interview (Peytchev, Peytcheva, and Groves 2010), while a later study found no association between response propensity and abortion reporting (Peytchev 2012). More research on this topic as well as further consideration of the influence on reporting of incentives and other approaches to improve response rates would be valuable.

### Survey Design

This analysis also points to the influence on reporting of other aspects of the survey design beyond the privacy afforded by the ACASI, such as length of recall. For each pregnancy outcome, the reporting ratio was smaller when a wider comparison period was used, highlighting some of the cognitive challenges of accurately reporting specific dates of pregnancy events. Only in the later surveys, when a five year recall period was used, were there differences by age in the patterns of reporting abortion; older women may find accurate recall over their longer fertility history more difficult. There is also confounding between the effects of recall period and time, which we are unable to fully distinguish in our analysis. Conceptually, however, it seems likely that both play a role in the reporting of abortion and miscarriage. In contrast, there were only modest differences in reporting of births between survey modes, and these differences appear to be primarily due to issues of misdating of events given the small expanded reporting ratios. Additionally, some women appear to have not followed the cue to report pregnancies only in the last five years in the ACASI, highlighting that questions wording also is an important influence on reporting quality.

There are two ways by which the format of the pregnancy history module might influence reporting of pregnancy outcomes. Firstly, a pregnancy history may enable women to omit, or choose to omit, certain pregnancies—either because they are less salient, or because they do not wish to talk about them. This would seem more likely for abortions and miscarriages than for births. A direct question gives less room for such interpretation. Second, the length and detail of the pregnancy history itself may discourage reporting, especially of abortions and miscarriages. The FTF interview is longer and more burdensome to complete than the parallel measures in the ACASI. For example, in the FTF interview a woman reporting a pregnancy is asked a minimum of 11 follow‐up questions for each pregnancy ending in live birth and six for an abortion. In contrast, the ACASI includes only a single question about each outcome and does not have any follow‐up questions for these items. Respondents may anticipate (or learn during the interview process) that by reporting fewer pregnancies in the FTF interview they will be required to answer fewer questions, and so may omit some of their pregnancies in this section in order to shorten the length of the interview. (Miscarriages and abortions are easier to omit than live births because there is no child in the household roster). Analyses of the British National Survey of Sexual Attitudes and Lifestyles Survey (NATSAL) found that abortion reporting declined after a change in the measurement of abortion from a direct question (ever had an abortion) to its inclusion as part of a more complicated pregnancy history (Copas et al. 2002; Wadsworth et al. 1993; Scott et al. 2017). Additionally, a recent study of the Demographic and Health Surveys (DHS) found that longer and more complicated surveys resulted in less complete reporting of births (Bradley 2015). Future research might consider the potential value of using more simplified approaches to data collection.

### Research Implications

These findings suggest that analysts must be extremely wary of using the pregnancy history data in the NSFG. Currently, the NSFG User Guide warns that the abortion measures in the FTF interview “should **not** be used for substantive research focused on the determinants or consequences of abortion” (National Center for Health Statistics 2004). Given the evidence found here of the sensitivity of reporting of miscarriages, a similar caution may be appropriate for miscarriages as well. The patterns of reporting have implications for research in at least two ways. First, levels of these outcomes or any outcomes that depend on them (such as overall pregnancy) will be underestimated. Less recognized and of more concern however, is that because of differential reporting by sociodemographic characteristics, any associations with other characteristics, including the causes and consequences of pregnancy, will be likely biased or incorrect. Research studies which use abortion data in these surveys, particularly from the FTF interviews, often overlook these data quality concerns and implications (Cougle, Reardon, and Coleman 2005; Reeves and Venator 2015), as do studies of miscarriage and pregnancy experiences more generally (Ahrens et al. 2018; K. Gold, Sen, and Hayward 2010).

We do not know that the greater number of women reporting an abortion in the ACASI than the FTF interview is a more accurate measure. Our expanded reporting ratio, which contrasts the five year reporting in the ACASI with six years in the FTF pregnancy history, suggests that some of the increased reporting in the ACASI is from measurement error from misdating. Additionally, our sensitivity analyses suggested that in many cases, women appear to be reporting their lifetime number of abortions in the ACASI when asked to report on events in the last five years. This would indicate a comprehension problem with the ACASI questions that has not yet been well identified or investigated. Future research might use cognitive interviewing techniques to better understand how respondents interpret and answer the questions. Given the observed patterns of reporting in this analysis, we propose that the NSFG might collect more reliable and accurate data if they were to return to asking lifetime measures in the ACASI, as opposed to a narrower recall window which seems to result in misdating of events or even a fundamental misunderstanding of the ACASI questions.

High quality research depends on unbiased data obtained from survey respondents. However, social stigma surrounding behaviors or experiences means that individuals may be reluctant to disclose their participation in those behaviors or experiences. Whether surveys can be designed to provide sufficient privacy and confidentiality to allow for full disclosure when respondents perceive strong social disapproval remains an open question. Our findings, alongside those of other studies examining women's experiences of abortion stigma, highlight the need to recognize that social stigma likely affects different segments of the population in different ways. For some women, the appearance of confidentiality may not be enough to induce reporting of a pregnancy event they feel is stigmatized.

While this research focuses narrowly on pregnancy outcomes, it may have implications for the measurement of other sensitive behaviors. Our findings support the hypothesis that a key mechanism of the ACASI is to increase privacy, and thus reduce the stigma felt by the respondent in reporting a sensitive outcome or behavior. Documenting changes over time, these findings indicate that the usefulness of ACASI varies in relationship to the social context, and its relative effectiveness should not be assumed to be static. Attention also needs to be paid to other cognitive demands posed by the interview questions. Our findings suggest that survey level factors such as the effort and time demanded of the respondent, and recall of events, may also influence the reporting of sensitive behaviors and that this may vary for different behaviors. More methodological research to explicitly test the interaction of these other influences on ACASI measures across a range of sensitive behaviors would be valuable for understanding ways to improve reporting.

### Strengths and Limitations

A key strength of this study is that we are able to use pregnancy outcome measures from two sections of the same survey, comparing responses between the same respondents in one section and another. Furthermore, we can compare reporting patterns between pregnancy outcomes that are asked in both sections to disentangle, to some extent, the contributions of stigma and other survey factors.

This study has a number of limitations. Although this analysis has highlighted the key role of the ACASI in eliciting disclosures of abortion from more women, some women who have had abortions will not report this in either mode. The extent and correlates of this type of underreporting cannot be identified with the current analytical approach. Second, our analytical approach of focusing on aggregated reporting ratios means that we can only examine bivariate associations across demographics; these reporting ratios cannot be included in multivariate models. Further research which examines multivariate influences on abortion stigma would be useful. Third, we cannot fully isolate the influence of the ACASI as a survey mode from other survey design factors which may have affected reporting and more experimental research is needed in this area. In particular, we cannot fully disentangle survey question effects related from to length of recall from time period effects. A further aspect of reporting that we cannot be sure about in our results is whether women are misreporting their abortions as miscarriages. This may be particularly relevant in more recent years, since the wider availability of medical abortion, whereby women are able to induce an abortion by taking two pills rather than be admitted for a surgical procedure (R. Jones and Jerman 2014). The abortion itself then usually takes place at home, and is more similar to a miscarriage than a surgical abortion. Whilst it is possible that this may result in abortions being misreported as miscarriages, the wording around abortion does not change between the FTF and ACASI survey modules, and so we would expect this to affect responses to both survey modes, and therefore not affect the reporting ratios. Finally, inherent in the NSFG survey design is that all respondents receive the ACASI module after the pregnancy history module in the FTF interview. This may influence their responses to the ACASI and FTF interviews in ways that we cannot measure in this analysis, for example if these topics become more salient when they are asked for a second time.

The increased reporting of abortion observed in the ACASI as compared to the FTF interview in the NSFG may not extend to other settings. While there has been an increase in the use of computer‐administered modes in developing countries, this is still not the norm (for example the Demographic and Health Surveys rely on interview administered surveys (Langhaug, Sherr, and Cowan 2010)). New technologies such as smartphones and Open Data Kit (ODK) software are allowing for increases in computer assisted interviewing in new settings and driving innovation (Zimmerman et al. 2017). Still, in countries in which abortion is largely illegal or clandestine, efforts to obtain abortion incidence measures have often relied on indirect estimation; the Abortion Incidence Complications Methodology (AICM) is widely used (recent examples include studies in Senegal (Sedgh et al. 2015) and Nepal (Puri et al. 2016)). Indirect methods which ask women to report on others in their network, such as the best‐friend approach and anonymous third‐party reporting are also being explored with mixed results (Scott et al. 2018; Sedgh et al. 2011; Yeatman and Trinitapoli 2011). Direct reporting approaches have included methods such as the sealed envelope method to measure abortion in Philippines (Juarez, Cabigon, and Singh 2010), Nigeria, and Zambia (Biddlecom et al. 2012), and list experiments in to measure abortion incidence in Liberia (Moseson et al. 2015), Vietnam (Moseson, Treleaven, et al. 2017), and Rajasthan, India (Bell and Bishai 2018), as well as in the US (Moseson, Gerdts, et al. 2017); these studies have inconsistent results.

Other approaches to improve measurement and the study of abortion are also being developed. The development of new survey questions designed to reduce the sensitivity of abortion have thus far had very modest success (R. Jones, Jerman, and Ingerick 2018; Kopplin, Desai, and Lindberg 2017), but should be explored further. Similarly, efforts to statistically model abortion reporting have thus far produced weak results that are highly sensitive to alternative specification (Tennekoon 2017; Tierney 2017; Yan, Kreuter, and Tourangeau 2012). Survey approaches focusing specifically on sampling women obtaining abortion, rather than the general population, also offer ways to learn about women's experiences of abortion without the same types of measurement error. The Abortion Patient Survey, conducted by the Guttmacher Institute, collects data on women obtaining abortions at a nationally representative sample of health facilities in the US (R. Jones, Jerman, and Ingerick 2018). The Turnaway Study is a prospective longitudinal study following US women who were and were not able to obtain the abortions they presented for (Foster et al. 2015). Both of these studies have allowed for important research on the characteristics and experiences of women having abortions (Biggs et al. 2017; R. Jones and Jerman 2017).

Good data on abortion is key to research on all pregnancy outcomes. It is also difficult to obtain. However, this analysis has shown some ways in which reporting of abortions is modified by methodological factors, and suggests that reporting can be improved. Continued investment into improving reporting is therefore a valuable and necessary contribution to future research on all reproductive outcomes.

## Supporting information

Appendix 1: Ratios of number of women reporting abortions, miscarriages, and births in the last five years in ACASI to Face‐to‐Face modes, by women's characteristics: 2006‐2010 National Survey of Family Growth
Appendix 2: Ratios of number of women reporting abortions, miscarriages, and births in the last five years in ACASI to Face‐to‐Face modes, by women's characteristics: 2006‐2010 National Survey of Family Growth: 2002 National Survey of Family Growth

## Footnotes

1 Other large national surveys in the United States currently using ACASI include the National Longitudinal Survey of Youth, National Study on Drug Use and Health, the National Health and Nutrition Examination Survey, and the National Longitudinal Study of Adolescent and Adult Health. Examples of surveys outside of the United States that use an ACASI include the National Survey of Sexual Attitudes and Lifestyles in Britain and surveys in other diverse settings (Falb et al. 2017; Gorbach et al. 2013).
