# Supplementary material for: Effect of ACASI on Reporting of Abortion and Other Pregnancy Outcomes in the US National Survey of Family Growth
Source: Stud Fam Plann. 2018 Jul 24;49(3):259–78. doi: 10.1111/sifp.12068 (PMC6166437; doi:10.1111/sifp.12068)
Supplement: Supplementary file 1 — Appendix 1: Ratios of number of women reporting abortions, miscarriages, and births in the last five years in ACASI to Face‐to‐Face modes, by women's characteristics: 2006‐2010 National Survey of Family Growth Appendix 2: Ratios of number of women reporting abortions, miscarriages, and births in the last five years in ACASI to Face‐to‐Face modes, by women's characteristics: 2006‐2010 National Survey of Family Growth: 2002 National Survey of Family Growth [file SIFP-49-259-s001.docx]

| **Appendix 1: Ratios of number of women reporting abortions, miscarriages, and births in the last five years in ACASI to Face-to-Face modes, by women’s characteristics: 2006-2010 National Survey of Family Growth** | | | | | | | | |
| --- | --- | --- | --- | --- | --- | --- | --- | --- |
|  | **Abortions** | |  | **Miscarriages** | |  | **Births** | |
|  | **Ratio** | **95% CI** |  | **Ratio** | **95% CI** |  | **Ratio** | **95% CI** |
| **Total** | 1.32 | (1.26-1.38) |  | 1.41 | (1.37-1.45) |  | 1.10 | (1.09-1.12) |
| **Age group** |  |  |  |  |  |  |  |  |
| 15-24 | 1.14* | (1.08-1.20) |  | 1.36 | (1.28-1.44) |  | 1.01* | (0.99-1.03) |
| 25-34 *(ref)* | 1.31 | (1.21-1.41) |  | 1.42 | (1.36-1.49) |  | 1.07 | (1.05-1.08) |
| 35-44 | 2.05* | (1.71-2.39) |  | 1.44 | (1.35-1.54) |  | 1.30* | (1.25-1.34) |
| **Race/Ethnicity** |  |  |  |  |  |  |  |  |
| White (Non-Latina) *(ref)* | 1.21 | (1.13-1.29) |  | 1.22 | (1.17-1.28) |  | 1.07 | (1.05-1.09) |
| Black (Non-Latina) | 1.21 | (1.14-1.28) |  | 1.60* | (1.50-1.70) |  | 1.17* | (1.14-1.20) |
| Other (Non-Latina) | 1.39 | (1.14-1.64) |  | 1.54* | (1.29-1.79) |  | 1.14* | (1.09-1.19) |
| Latina | 1.74* | (1.54-1.95) |  | 1.56* | (1.45-1.67) |  | 1.09 | (1.07-1.11) |
| **Poverty level income** |  |  |  |  |  |  |  |  |
| 0-99% | 1.39* | (1.28-1.49) |  | 1.65* | (1.53-1.77) |  | 1.10* | (1.08-1.12) |
| 100-299% | 1.31 | (1.21-1.41) |  | 1.38* | (1.31-1.44) |  | 1.12* | (1.10-1.14) |
| 300%> *(ref)* | 1.25 | (1.15-1.34) |  | 1.17 | (1.11-1.23) |  | 1.08 | (1.06-1.10) |
| **Marital status** |  |  |  |  |  |  |  |  |
| Currently married *(ref)* | 1.93 | (1.62-2.23) |  | 1.26 | (1.20-1.31) |  | 1.08 | (1.06-1.09) |
| Previously married | 1.46* | (1.26-1.67) |  | 1.57* | (1.44-1.71) |  | 1.21* | (1.17-1.26) |
| Never married | 1.19* | (1.14-1.24) |  | 1.50* | (1.43-1.58) |  | 1.10* | (1.08-1.12) |
| **Religious service attendance** | |  |  |  |  |  |  |  |
| Less than once a month/ Never *(ref)* | 1.19 | (1.12-1.25) |  | 1.40 | (1.34-1.45) |  | 1.09 | (1.07-1.11) |
| Once a month or more | 1.60* | (1.44-1.76) |  | 1.42 | (1.36-1.49) |  | 1.12* | (1.10-1.14) |
| * p < 0.05 (ref) indicates the reference category for each variable | | | | |  |  |  |  |
|  |  |  |  |  |  |  |  |  |

| **Appendix 2: Ratios of number of women reporting abortions, miscarriages, and births in the last five years in ACASI to Face-to-Face modes, by women’s characteristics: 2006-2010 National Survey of Family Growth: 2002 National Survey of Family Growth** | | | | | | | | |
| --- | --- | --- | --- | --- | --- | --- | --- | --- |
|  | **Abortions** | |  | **Miscarriages** | |  | **Births** | |
|  | **Ratio** | **95% CI** |  | **Ratio** | **95% CI** |  | **Ratio** | **95% CI** |
| **Total** | 1.14 | (1.13-1.16) |  | 1.12 | (1.11-1.14) |  | 1.00 | (1.00-1.01) |
| **Age group** |  |  |  |  |  |  |  |  |
| 15-24 | 1.16 | (1.11-1.22) |  | 1.21* | (1.17-1.26) |  | 1.03* | (1.01-1.04) |
| 25-34 *(ref)* | 1.14 | (1.11-1.16) |  | 1.11 | (1.08-1.14) |  | 1.00 | (0.99-1.00) |
| 35-44 | 1.14 | (1.11-1.17) |  | 1.10 | (1.09-1.12) |  | 1.00 | (0.99-1.00) |
| **Race/Ethnicity** |  |  |  |  |  |  |  |  |
| White (Non-Latina) *(ref)* | 1.09 | (1.06-1.11) |  | 1.06 | (1.04-1.07) |  | 1.00 | (1.00-1.01) |
| Black (Non-Latina) | 1.15* | (1.12-1.18) |  | 1.20* | (1.15-1.26) |  | 1.01 | (1.00-1.01) |
| Other (Non-Latina) | 1.20* | (1.11-1.30) |  | 1.15* | (1.06-1.23) |  | 1.02^ | (1.00-1.04) |
| Latina | 1.27* | (1.20-1.35) |  | 1.18* | (1.14-1.22) |  | 1.00* | (0.99-1.00) |
| **Poverty level income** |  |  |  |  |  |  |  |  |
| 0-99% | 1.20* | (1.15-1.25) |  | 1.22* | (1.18-1.25) |  | 1.01 | (1.00-1.01) |
| 100-299% | 1.15* | (1.12-1.17) |  | 1.10* | (1.08-1.13) |  | 1.00* | (0.99-1.00) |
| 300%> *(ref)* | 1.11 | (1.09-1.14) |  | 1.08 | (1.06-1.10) |  | 1.01 | (1.00-1.01) |
| **Marital status** |  |  |  |  |  |  |  |  |
| Currently married *(ref)* | 1.15 | (1.12-1.18) |  | 1.08 | (1.06-1.10) |  | 1.00 | (0.99-1.00) |
| Previously married | 1.11* | (1.07-1.14) |  | 1.10 | (1.07-1.13) |  | 1.01* | (1.00-1.01) |
| Never married | 1.16 | (1.13-1.19) |  | 1.21* | (1.17-1.25) |  | 1.02* | (1.01-1.03) |
| **Religious service attendance** |  |  |  |  |  |  |  |  |
| Less than once a month/ Never *(ref)* | 1.11 | (1.09-1.13) |  | 1.11 | (1.09-1.13) |  | 1.01 | (1.00-1.01) |
| Once a month or more | 1.20* | (1.17-1.23) |  | 1.13 | (1.11-1.15) |  | 1.00^ | (1.00-1.01) |
| * p < 0.05 (ref) indicates the reference category for each variable | | | | | | | | |
